# A Comparison of Classical Force-Fields for Molecular Dynamics Simulations of Lubricants

**DOI:** 10.3390/ma9080651

**Published:** 2016-08-02

**Authors:** James P. Ewen, Chiara Gattinoni, Foram M. Thakkar, Neal Morgan, Hugh A. Spikes, Daniele Dini

**Affiliations:** 1 Department of Mechanical Engineering, Imperial College London, South Kensington Campus, Exhibition Road, London SW7 2AZ, UK; c.gattinoni@imperial.ac.uk (C.G.); h.spikes@imperial.ac.uk (H.A.S.); d.dini@imperial.ac.uk (D.D.); 2 Shell India Markets Private Limited, 8B RMZ Centennial Building, Kundanahalli Main Road, Bangalore 560048, India; foram.thakkar@shell.com; 3 Shell Global Solutions UK Ltd., Brabazon House, Manchester M22 0RR, UK; n.morgan@shell.com

**Keywords:** tribology, molecular dynamics, force-fields, lubricants

## Abstract

For the successful development and application of lubricants, a full understanding of their complex nanoscale behavior under a wide range of external conditions is required, but this is difficult to obtain experimentally. Nonequilibrium molecular dynamics (NEMD) simulations can be used to yield unique insights into the atomic-scale structure and friction of lubricants and additives; however, the accuracy of the results depend on the chosen force-field. In this study, we demonstrate that the use of an accurate, all-atom force-field is critical in order to; (i) accurately predict important properties of long-chain, linear molecules; and (ii) reproduce experimental friction behavior of multi-component tribological systems. In particular, we focus on *n*-hexadecane, an important model lubricant with a wide range of industrial applications. Moreover, simulating conditions common in tribological systems, i.e., high temperatures and pressures (HTHP), allows the limits of the selected force-fields to be tested. In the first section, a large number of united-atom and all-atom force-fields are benchmarked in terms of their density and viscosity prediction accuracy of *n*-hexadecane using equilibrium molecular dynamics (EMD) simulations at ambient and HTHP conditions. Whilst united-atom force-fields accurately reproduce experimental density, the viscosity is significantly under-predicted compared to all-atom force-fields and experiments. Moreover, some all-atom force-fields yield elevated melting points, leading to significant overestimation of both the density and viscosity. In the second section, the most accurate united-atom and all-atom force-field are compared in confined NEMD simulations which probe the structure and friction of stearic acid adsorbed on iron oxide and separated by a thin layer of *n*-hexadecane. The united-atom force-field provides an accurate representation of the structure of the confined stearic acid film; however, friction coefficients are consistently under-predicted and the friction-coverage and friction-velocity behavior deviates from that observed using all-atom force-fields and experimentally. This has important implications regarding force-field selection for NEMD simulations of systems containing long-chain, linear molecules; specifically, it is recommended that accurate all-atom potentials, such as L-OPLS-AA, are employed.

## 1. Introduction

Molecular dynamics simulations using both *ab initio* and empirically-parameterized classical force-fields can accurately reproduce important properties of molecular systems under ambient conditions. However, they are generally poorly transferable and their accuracy decreases when the system, conditions, or properties of interest deviate from those for which they were directly parameterized. Simulations of tribological systems are particularly demanding, since most lubricants contain relatively large, complex molecules, and the conditions of interest are typically high temperatures and high pressures (HTHP). This means that force-fields should ideally be tested for these molecules and conditions prior to their application in complex simulations. Despite these challenges, nonequilibrium molecular dynamics (NEMD) simulations using classical force-fields have shed light on the nanoscale behavior of many experimentally-important lubricant systems. These have included simulations investigating the rheological and tribological behavior of several base oils [1,2,3,4,5] as well as many important additives [6,7,8,9,10].

One of the most critical considerations when selecting a force-field for long-chain molecules is whether to employ an all-atom or a united-atom variant. In all-atom (or explicit hydrogen) force-fields, every atom in the molecule is explicitly treated and parameterized. On the contrary, united-atom force-fields do not explicitly treat non-polar hydrogen atoms, but rather group them with carbon atoms and assign parameters to the resultant CH, CH_2_, or CH_3_ ‘pseudo-atoms’. United-atom force-fields significantly reduce the number of atoms for which non-bonded interactions need to be calculated, thus decreasing the computational expense by up to an order of magnitude [11]. This has made them very popular in tribology [1,2,3,4,5,6,7] where the simulation of large, multicomponent systems is often required.

Currently, it is largely unclear which force-fields will yield the most accurate results in the of context tribology. Probably the most significant liquid properties in the simulation of tribological systems are the density and viscosity, since they are inherently linked to the lubricant hydrodynamics. In an MD force-field, the liquid density is mainly governed by the non-bonded (Lennard-Jones and Coulombic) interactions, whilst the viscosity is also heavily influenced by the ‘stiffness’ of the torsional potential [12,13]. Previous studies have compared all-atom and united-atom force-field performance in terms of their density and viscosity prediction of long-chain alkanes [13,14,15]; however, they have been limited to only a few variants, and have not included recent parameterizations specifically designed for long-chain molecules [16,17]. These previous comparisons have shown that united-atom force-fields consistently under-predict the viscosity of long-chain linear alkanes, with the prediction accuracy deteriorating when longer chain molecules or high pressures are used [13,14,15]. For example, under ambient conditions, united-atom force-fields have been shown to provide a reasonable prediction for the viscosity of C_10_ (−20%); however, prediction accuracy decreases considerably for longer C_16_ molecules (−50%) [15]. Although improvements to the prediction of transport properties by united-atom force-fields have been made by using modified forms of non-bonded interaction (other than Lennard-Jones), such changes can often have a detrimental effect on the thermodynamic properties for which the force-fields were originally parameterized [18]. It has also been shown that many popular all-atom force-fields yield a much higher melting point for long-chain alkanes than the experimental value, which in turn leads to drastically elevated density and viscosity values [16,17,19,20]. This may be critical in simulations of confined systems, where intricate phase transitions can heavily influence the tribological behavior observed [4,21,22]. In summary, viscosity and melting point under- and over-prediction respectively will both considerably impact the simulated behavior of long-chain molecules, which are of particular importance in tribology. Indeed, the results of several previous MD simulations of dense, multicomponent systems have suggested that the use of accurate all-atom force-fields is critical in obtaining accurate structure and friction results [22,23,24,25,26,27,28].

This two-part study draws attention to issues with several popular classical force-fields for MD simulations of long-chain linear alkanes, and highlights contemporary alternatives which provide far higher accuracy. The simulation of these molecules of are great interest due to their prevalence in fuels and lubricants as well as biological systems. The first section (2.2, 3.2) involves force-field benchmarking of many popular force-fields, including recent parameterizations for long-chain molecules, in terms of their ability to predict the density and viscosity of *n*-hexadecane in equilibrium molecular dynamics (EMD) simulations. These properties are significant since they govern the hydrodynamics of lubricants and directly influence their tribological behavior. In the second section (2.3, 3.3), the most accurate all-atom and united-atom force-field from the first section are compared in confined NEMD simulations of a complex tribological system. Specifically, the structure and friction of stearic acid films adsorbed on iron oxide surfaces and separated by a thin layer of *n*-hexadecane are compared between when united-atom and all-atom force-fields are used, as well as with experiments.

## 2. Methodology

All systems were constructed using the Materials and Processes Simulations Platform (MAPS) from Scienomics SARL (Paris, France) and MD simulations were performed in LAMMPS [29]. The MD equations of motion were integrated using the velocity-Verlet algorithm with an integration time-step of 1.0 fs for all of the force-fields. Fast-moving bonds involving hydrogen atoms were constrained with the SHAKE algorithm [30]. The specific methodologies for the two sections will now be described separately.

### 2.1. Density and Viscosity Benchmarking

#### 2.1.1. Setup

In this section, many classical force-fields are benchmarked in terms of their density and viscosity prediction of *n*-hexadecane. The Newtonian shear viscosity is calculated using the Green-Kubo formula [31,32] applied to classical EMD simulations. Since the Green-Kubo equations allow a direct route to the Newtonian shear viscosity, it is probably the most suitable method to benchmark the considerable number of force-fields used here. Issues regarding slow convergence of the running average viscosity [33] and the potential for systems to become trapped in local minima [15] have been encountered when using this method for long-chain molecules. However, by monitoring the ensemble average viscosity over multiple-trajectories, as suggested by Payal et al. [15], and utilized here, the impact of both of these problems can be minimized. The selection of *n*-hexadecane as a model lubricant is justified by its extensive use in experimental and modelling tribology studies, as well as the fact that its density and viscosity are well-characterized under a range of experimental conditions. The conditions simulated in this section range between ambient (300 K, 0.1 MPa) and HTHP (423 K, 60.8 MPa; 423 K, 202.7 MPa) conditions [15], which are of direct relevance to tribological systems.

The force-fields selected for the benchmarking simulations are those developed by: Chynoweth and Michopoulos (CM-UA) [34], Nath et al. (NERD-UA) [35], Schuler et al. (GROMOS-UA) [36], Martin et al. (TraPPE-UA, 12-6) [11], Potoff and Kamath (TraPPE-UA, 16-6) [37], Mayo et al. (Dreiding-AA) [38], Cornell et al. AMBER-AA [39], Jorgensen et al. (OPLS-AA) [40], Murzyn et al. (P-OPLS-AA) [16] and Siu et al. (L-OPLS-AA) [17]. The overall functional form of these classical force-fields are quite similar (Equation (1)), with all including terms for bond stretching, bending and torsion (bonded), as well as Van der Waals and Coulombic interactions (non-bonded). However, each contains unique force-field parameters and many have different forms of non-bonded and torsional potentials, non-bonded mixing rules, 1-4 non-bonded scaling factors and some utilize Coulombic parameters for alkyl C and H atoms whilst others do not.

(1) $$E_{Total}=E_{Bond}+\;E_{Angle}+E_{Torsion}+E_{L-J}+E_{Coulomb}$$

**Figure 1:**
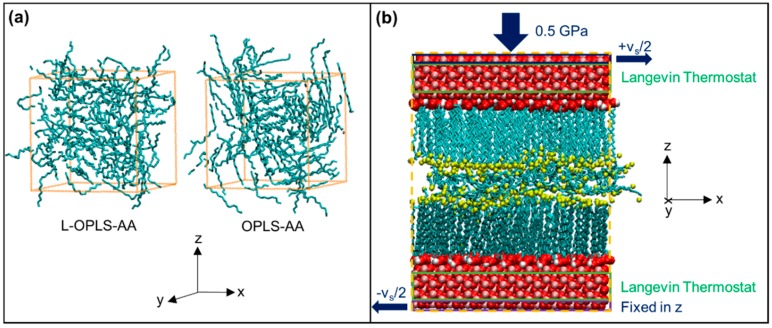
Simulation details: setup for EMD (**a**) and NEMD (**b**) simulations. EMD shows equilibrated systems after 2.0 ns NPT phase (300 K, 0.1 MPa) when using OPLS (liquid) and L-OPLS (crystallized). NEMD example shown for all-atom at high coverage after compression, before sliding. Terminal C atoms are yellow (in b), chain C atoms are cyan, O atoms are red, H atoms are white and Fe atoms are pink. Non-polar hydrogen atoms in the tail groups are not shown for clarity. Periodic boundary conditions (orange dotted line) applied in all directions in a, *x* and *y* in b. Rendered using VMD [46].

A box of 130 *n*-hexadecane molecules was generated and periodic boundary conditions were applied in all directions (Figure 1a). The cut-off radius for Lennard-Jones interactions was fixed at 2.5σ (≈13 Å). Long-range tail corrections to the energy and pressure were included [41]. ‘Unlike’ interactions were evaluated using the either the Lorentz—Berthelot or geometric mean mixing rules, depending on the force-field. Long-range electrostatic interactions were not cut off, but were evaluated using the PPPM method [42]. The Nosé–Hoover thermostat and barostat [43,44,45], with relaxation constants of 0.1 and 1.0 ps, respectively, were employed to maintain the desired temperature and pressure.

#### 2.1.2. Procedure

**Figure 2:**
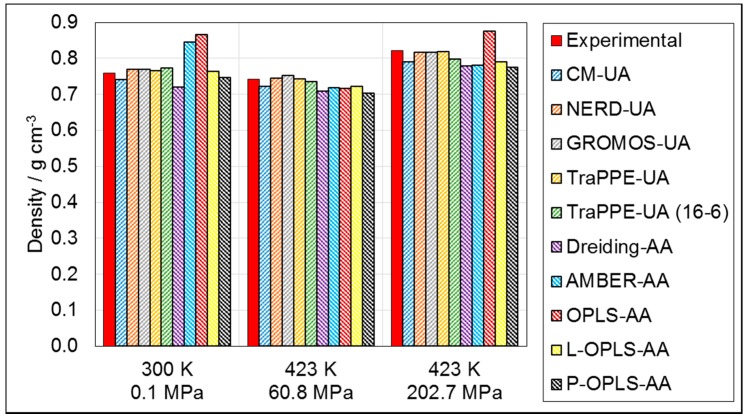
Simulated density (NPT) for *n*-hexadecane at; 300 K, 0.1 MPa; 423 K, 60.8 MPa; and 423 K, 202.7 MPa. United-atom force-fields represented by forward dashes, all-atom force-fields represented by backwards dashes. Experimental data are reproduced with permission from ref. [15].

First, the system was energy minimized before being equilibrated in the isothermal-isobaric (NPT) ensemble until there was only negligible change in the density (2.0–10.0 ns). The density values shown in Figure 2 were obtained from the average volumes of the simulation boxes during the final 1.0 ns of these NPT simulations. The simulation box volume was then fixed in order to match this average density and the system was equilibrated in the canonical ensemble (NVT) for a period of 2.0 ns. From this equilibrated system, five independent trajectories were produced through separate heat-quench cycles. For the 300 K and 0.1 MPa simulations, the first part of the cycle consisted of 1.0 ns NVT simulations at *T* = 305, 310, 315, 320, and 325 K, after which the systems were quenched back to 300 K during another 1.0 ns NVT simulation. The viscosity was then calculated from subsequent 3.0 ns NVT production runs, starting from the five independent trajectories. The Newtonian shear viscosity, η, was evaluated using the Green-Kubo [31,32] formula:

(2) $$\eta =\frac{V}{k_{B}T}\int_{0}^{\infty }dt\langle P_{\alpha \beta }\left(t_{0}\right)P_{\alpha \beta }{(t+t_{0})\rangle }_{t_{0}}$$

where $\langle P_{\alpha \beta }\left(t_{0}\right)P_{\alpha \beta }\left(t+t_{0}\right)\rangle$ is the stress autocorrelation function (ACF) for the off-diagonal components of the stress tensor $P_{\alpha \beta }$, with α and β representing the *x*, *y*, or *z* direction in Cartesian coordinates. The running integral of the stress ACF yields the Newtonian shear viscosity (Equation (2)). V is the volume of the simulation box, $k_{B}$ is the Boltzmann constant, T is the temperature. For practical reasons, the upper limit in numerical calculations of the integral is set to a certain time, *t*_max_, which is sufficiently long to ensure the noise-free decay of ACF, which is calculated for a limited number of consecutive time origins, *t*_0_ [16]. Generally, $t_{max}$ should be long enough in order to ensure convergence of the viscosity by exceeding the rotational relaxation time of the molecule [33]. It should be noted that the rotational relaxation time, required integration time and ultimately the simulation length, increases by more than an order of magnitude with a relatively modest increase in chain-length (C_10_ to C_16_) [33]. Therefore, it is suggested that other, less direct routes to the Newtonian shear viscosity, such as NEMD [1] or R-NEMD [47], are used for chain-lengths greater than C_16_. However, for *n*-hexadecane, the ensemble average Green-Kubo approach employed here allows sampling of a large configurational space and represents a relatively fast, accurate route to the Newtonian shear viscosity [15].

### 2.2. Structure and Friction in Multicomponent Tribological Systems

#### 2.2.1. Setup

The methodology for the second section is described briefly here and in more detail in ref. [10]. A representative example of the systems simulated in this study is shown in Figure 1b. It consists of a thin layer of *n*-hexadecane lubricant confined between two stearic acid monolayers adsorbed on iron oxide slabs. Stearic acid was chosen as a model organic friction modifier (OFM), which are an important class of boundary lubricant additive [10,48].

Two (100) slabs of α-iron(III)-oxide [49] (hematite) with dimensions (*xyz*) of approximately 55 Å × 55 Å × 12 Å were used as the substrates, representing a single asperity contact. Periodic boundary conditions were applied in the *x* and *y* directions. Stearic acid molecules were oriented perpendicular to, and initially 3 Å from, the interior surfaces of the two slabs (Figure 1b). Two horizontally-orientated molecular layers (70 molecules) of *n*-hexadecane were then randomly distributed between the stearic acid films. This thickness of *n*-hexadecane was determined from previous squeeze-out simulations using the same systems, as described in ref. [10].

Three coverages of stearic acid are considered; a high surface coverage (*Γ* = 4.32 nm^−2^) close to the maximum theoretical value; a medium coverage (*Γ* = 2.88 nm^−2^) approximately 2/3 of the maximum coverage; and a low coverage (*Γ* = 1.44 nm^−2^) around 1/3 of the maximum coverage. This corresponds to 132, 88, and 44 stearic acid molecules on each 30 nm^2^ slab respectively. The highest coverage simulated has been observed experimentally for stearic acid on iron oxide surfaces [50].

The *n*-hexadecane and stearic acid molecules were represented by either; (i) L-OPLS-AA [17,40] (all-atom); or (ii) TraPPE-UA [11,51] (united-atom) force-fields. Lennard-Jones interactions were cut-off at 10 Å and ‘unlike’ interactions were evaluated using either the geometric mean (L-OPLS-AA) or Lorentz—Berthelot (TraPPE-UA) mixing rules. Electrostatic interactions were evaluated using a slab implementation of the PPPM algorithm [52]. Surface-hexadecane and surface-stearic acid interactions were represented by the Lennard-Jones and Coulomb potentials; the Fe and O hematite parameters selected were developed by Berro et al. [7]. The hematite slab atoms were restrained in the corundum crystal structure by harmonic bonds between atoms within 3 Å. The force constant of these bonds was chosen to be 130 kcal·mol^−1^·Å^−2^, which has been shown previously to keep the surface structure suitably rigid, but not to adversely affect the thermostatting [9].

A temperature of 300 K is maintained using a Langevin thermostat [53], with a time relaxation constant of 0.1 ps. The pressure (*P_z_* = 0.5 GPa) was controlled by applying a constant normal force to the outermost layer of atoms in the upper slab, keeping the *z*-coordinates of the outermost layer of atoms in the lower slab fixed, as is common in confined NEMD simulations [6,7,8,9,10] (Figure 1b).

#### 2.2.2. Procedure

First, a density similar to that of liquid *n*-hexadecane (0.75 g·cm^−3^) was achieved by moving the top slab down at 10 m·s^−1^, prior to energy minimization. The system was then pressurized (*P_z_* = 0.5 GPa), thermostatted in the directions perpendicular to the compression (*x* and *y*), and allowed to equilibrate at 300 K. Initially, the slab separation varied in a damped harmonic manner, so sliding was not applied until a constant average slab separation was obtained and the hydrostatic pressure within the *n*-hexadecane film was close to its target value. These compression simulations were generally around 200 ps in duration.

After compressive oscillation became negligible, a velocity of *v*_x_ = ±*v*_s_/2 was added in the *x* direction to the outermost layer of atoms in each slab (Figure 1b) and sliding simulations were conducted for 0.5–10.0 ns, depending on the sliding velocity. The values of *v*_s_ applied were 1.0, 2.0, 5.0, 10.0, and 20.0 m·s^−1^ and all simulations were run for long enough to yield a sufficient sliding distance (10 nm) to obtain representative values for the friction coefficients (uncertainty < 10%). While lower sliding velocities are desirable to match those used in boundary friction experiments (typically mm·s^−1^), they are not yet accessible using NEMD simulations of this scale [9]. During the sliding simulations, any heat generated was dissipated using a thermostat acting only on the middle 10 Å of both iron oxide slabs (Figure 1b), applied in the direction perpendicular to the both the sliding and compression (*y*) [54]. This is known to be advantageous over direct thermostatting of the fluid, which has been shown to significantly affect the behavior of confined fluids under sliding conditions [55]. The boundary thermostatting method applied here has been shown previously to be effective in controlling the temperature of similar systems and sliding velocities [56]. At the onset of sliding, an expansion due to the increase of temperature by shear heating was expected so it was ensured that steady state sliding had been attained before sampling began for the friction coefficient [7]. The time taken to achieve steady state sliding decreased with increasing sliding velocity but always equated to approximately 2 nm of sliding distance.

The kinetic friction coefficient, μ, was obtained using the extended Amontons−Coulomb law under the high load approximation: F_L_/F_N_ ≃ μ. F_L_ and F_N_ are, respectively, the average total lateral and normal forces acting on the entire slab. The friction coefficients presented are the average of those calculated for the top and bottom slabs. The validity of the high load approximation has been confirmed from previous NEMD simulations of stearic acid layers with a thin, separating layer of *n*-hexadecane [8,9,10].

## 3. Results and Discussion

Results from the density and viscosity EMD benchmarking simulations are outlined first. This is followed by a comparison between all-atom and united-atom force-fields for the structure and friction of the stearic acid films at different coverages in NEMD simulations.

### 3.1. Density and Viscosity Benchmarking

#### 3.1.1. Density

Figure 2 shows that the density prediction for *n*-hexadecane was generally accurate (within 15% of experiment) for all of the force-fields and conditions tested. However, there were some important differences between the performances of the force-fields under ambient and HTHP conditions.

Dreiding-AA was designed as a ‘generic’ force-field and it was rather inaccurate in the density prediction of *n*-hexadecane, yielding a consistent under-prediction of approximately 5% under all of the conditions simulated. The initial parameterizations of the popular all-atom force-fields OPLS-AA and AMBER-AA over-predicted the density by 14% and 11%, respectively, at ambient conditions. This density over-prediction has been observed in previous simulations of long chain alkanes using these force-fields at ambient conditions and has been attributed to crystallization due to the over-prediction of the melting point (Figure 1a) [16,17,20]. Crystallization did not occur at 423 K and 60.8 MPa, and both OPLS-AA and AMBER-AA under-predicted the density by around 4%. AMBER-AA also under-predicted the density by around 4% at 423 K and 202.7 MPa, whereas OPLS-AA over-predicted the density by 7%, again suggesting crystallization. The forms of OPLS-AA with updated non-bonded and torsional parameters for long-chain molecules (P-OPLS-AA [16] and L-OPLS-AA [17]) were much more accurate. No crystallization was observed when P-OPLS-AA was used, and the density prediction at ambient conditions was significantly improved; however, a under-prediction of density at HTHP conditions was still observed. The *n*-hexadecane also remained liquid when L-OPLS-AA was used and density prediction was more accurate (within 5%) than for the other all-atom force-fields under all of the conditions simulated.

The density prediction using the united-atom force-fields was generally more accurate with all of them yielding a density value within 5% of the experimental one under all of the conditions simulated. This is probably the result of a more comprehensive choice of molecules during parameterization, including long-chain alkanes (up to C_32_) [11]. The version of TraPPE-UA using a 16-6 Mie Potential [37] for the Van der Waals interactions was found to be less accurate in terms of density prediction relative to the conventional 12-6 (Lennard-Jones) form [11].

In summary, the density is significantly overestimated when using the original parameterizations of some all-atom force-fields (OPLS-AA, AMBER-AA), which will considerably affect the behavior of long-chain alkanes in simulations. Other than these force-fields, density prediction is relatively accurate and the small variations are unlikely to have a significant effect on simulation results.

#### 3.1.2. Viscosity

**Figure 3:**
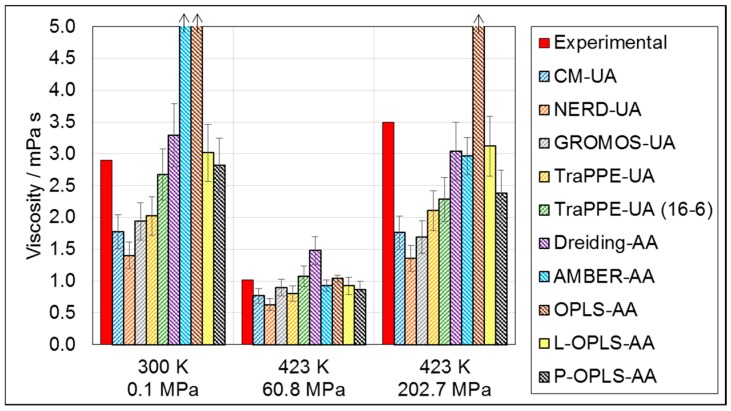
Simulated viscosity (NVT) for *n*-hexadecane at; 300 K, 0.1 MPa, 423 K, 60.8 MPa, and 423 K, 202.7 MPa. United-atom force-fields represented by forward dashes, all-atom force-fields represented by backwards dashes. Experimental data are reproduced with permission from [15]. Error bars show the standard deviation in the ensemble average viscosity between the five independent trajectories. Simulated viscosity bars are truncated and marked with an arrow when viscosity increased by over two orders of magnitude compared to experiment due to crystallization.

Figure 3 shows that viscosity prediction was much less accurate for all of the force-fields tested. This is expected since, unlike the density, the transport properties are not usually directly included in the parameterization of classical force-fields.

Given its relatively poor density prediction, the viscosity prediction of Dreiding-AA was surprisingly accurate, although it over-predicted viscosity by around 40% at 423 K and 60.8 MPa. OPLS-AA and AMBER-AA performed very poorly under ambient conditions, over-predicting the viscosity by more than two orders of magnitude, which can again be attributed to the crystallization of *n*-hexadecane (Figure 1a) [16,17,20]. AMBER-AA performed rather better at HTHP conditions than at ambient conditions; however, OPLS-AA still yielded severe viscosity over-prediction at 423 K and 202.7 MPa. The updated P-OPLS-AA [16] and L-OPLS-AA [17] include modified non-bonded and torsional parameters specifically for long-chain molecules. As a result, P-OPLS-AA accurately predicts the viscosity under ambient conditions and at 423 K and 60.2 MPa; however, at 423 K and 202.7 MPa, the viscosity is under-predicted by approximately 30%. Conversely, L-OPLS-AA accurately reproduces experimental viscosities (within 15%) under all of the conditions simulated. From the density and viscosity results, it is clear that L-OPLS-AA is the most accurate all-atom force-field with which to reproduce the density, viscosity and, thus, the hydrodynamics of the model base oil *n*-hexadecane. Therefore, it is taken forward to the all-atom/united-atom comparisons for NEMD simulations of tribological systems in the second section.

Viscosity prediction with the united-atom force-fields was generally rather inaccurate and a consistent under-prediction of between 10%–60% was observed depending on the conditions. Viscosity prediction was particularly poor at 423 K and 202.7 MPa, where the density is highest and the ‘roughness’ of individual molecules is likely to have a greater impact on the viscosity. The viscosity prediction of TraPPE-UA under ambient conditions was significantly improved through the use of a 16-6 non-bonded potential (−10% vs. −30%). However, the viscosity was still significantly under-predicted (−35%) at 423 K and 202.7 MPa. This suggests that whilst the physical presence of hydrogen atoms may not be essential in accurately predicting the viscosity of long-chain alkanes accurately at ambient conditions, they are more important at high pressures, where molecules are forced closer together leading to more steric clashes between hydrogen atoms on neighboring molecules. Although its viscosity prediction under ambient conditions was more accurate, the different functional form of the non-bonded interactions in the 16-6 version of TraPPE-UA makes it less compatible with other types of atoms, such as those in important functional groups or surfaces. Therefore, the standard 12-6 form of TraPPE-UA was selected for the all-atom/united-atom comparisons in the second section.

Generally, the density and viscosity prediction of the force-fields deteriorates only slightly when comparing ambient to HTHP conditions. Figure 2 and Figure 3 show that there is a tendency for force-fields to under-predict both density and viscosity under HTHP conditions, a factor which could be investigated further in the pursuit of force-fields specifically for simulations of long-chain molecules under HTHP conditions, which are of particular interest in tribology.

Viscosity over-prediction by the original parameterizations of popular all-atom force-fields (OPLS-AA, AMBER-AA) under ambient and HTHP conditions will have a substantial impact on the behavior of long-chain alkanes, and they should therefore be avoided in future simulations of these molecules. The viscosity under-prediction by all of the united-atom force-fields under ambient and HTHP conditions may also have a significant effect on simulation results. For example, in confined liquids, the friction coefficient depends greatly on the ability of individual molecules, molecular layers, and solid surfaces to slide past one another [4]. The significant under-prediction of the viscosity by united-atom force-fields suggests that molecules move past one another more easily than if all-atom force-fields are used, meaning that friction coefficients calculated for confined systems using united-atom force-fields will also be under-predicted; this effect will be explored in the second section.

### 3.2. Structure and Friction in Complex Tribological Systems

**Figure 4:**
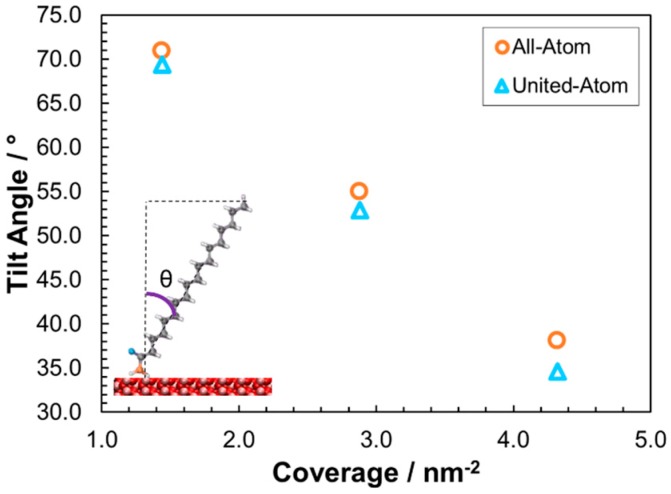
Variation in the average C_1–18_ tilt angle to surface normal, θ, as a function of coverage for all-atom and united-atom force-fields under sliding conditions, *P_z_* = 0.5 GPa, *v_s_* = 10 m·s^−1^.

**Figure 5:**
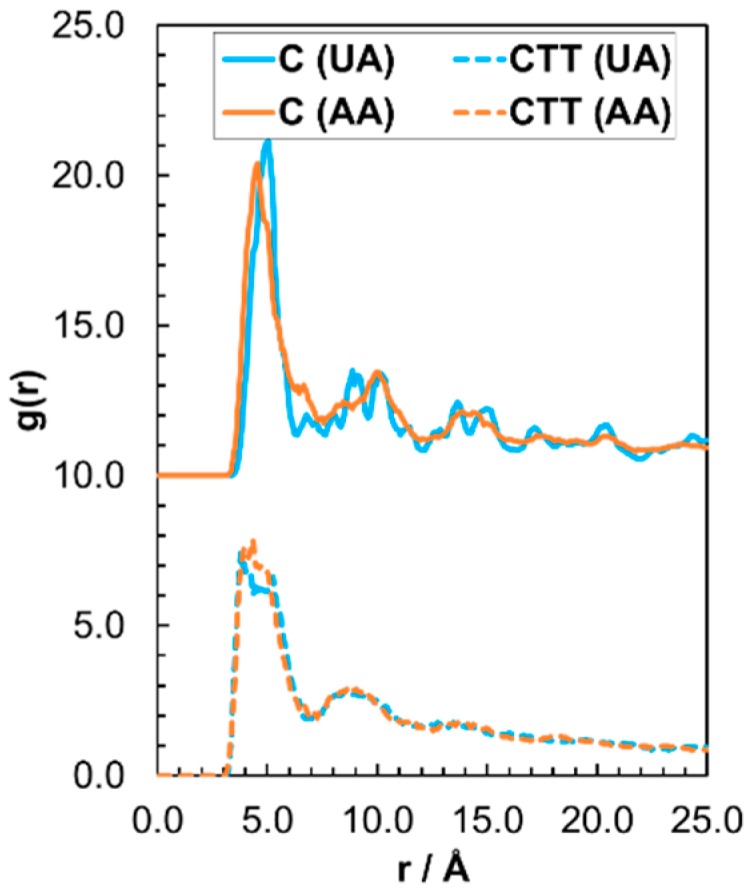
Radial distribution function (RDF), g(*r*), which describes the ordering of the terminal carbon atom (CTT) (dotted) and carbonyl C (solid) atoms for all-atom (orange) and united-atom (blue) at high coverage. The C RDFs are shifted upwards by 10 units for clarity.

**Figure 6:**
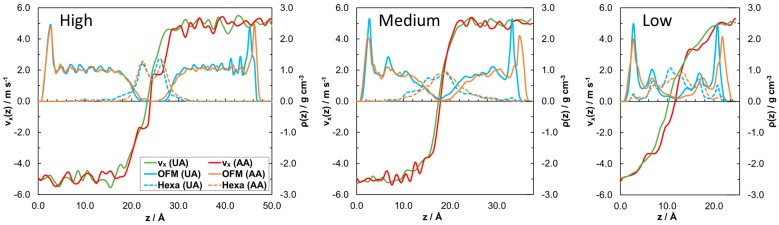
Profile of atom *x* velocities from 0.5 Å spatial bins in *z*, *v_x_*(*z*), overlaid on to atomic mass density profile in *z*, *ρ*(*z*), at high, medium, and low coverage. *P_z_* = 0.5 GPa, *v*_s_ = 10 m·s^−1^.

**Figure 7:**
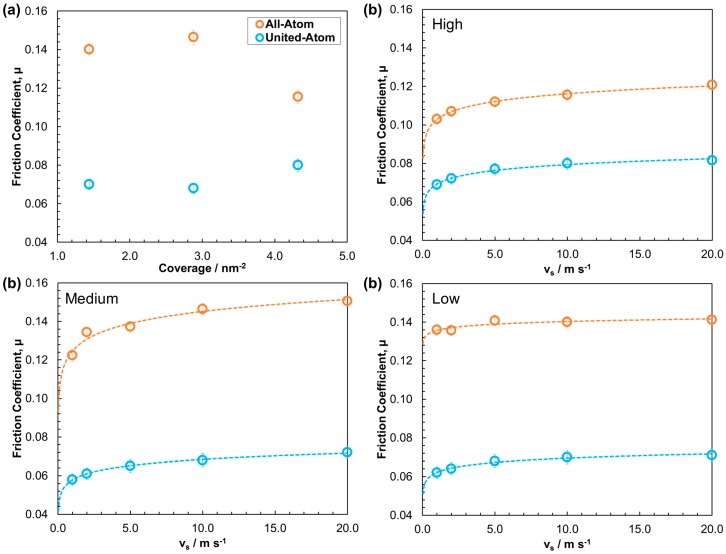
Friction coefficient: as a function of coverage at *v*_s_ = 10 m·s^−1^ (**a**) and as a function of sliding velocity (**b**) at high, medium, and low coverage. Dotted line is a logarithmic fit of the friction-velocity data. Error bars calculated from standard deviation between block averages from 100 ps time windows.

**Figure 8:**
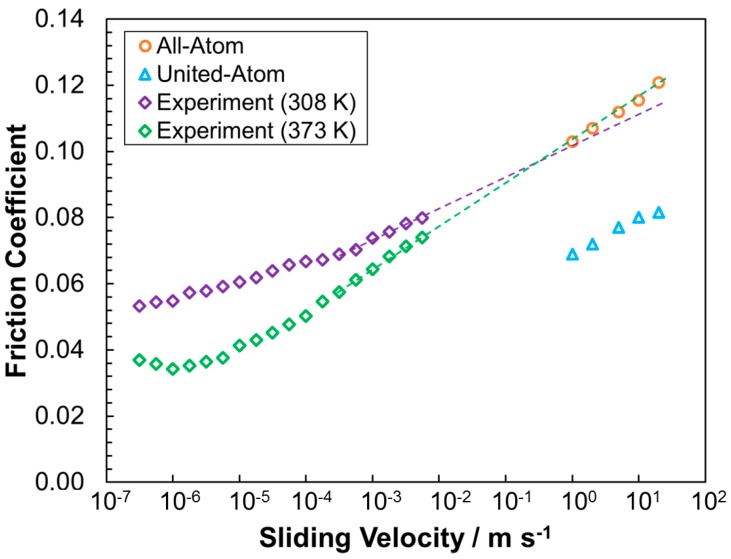
Variation in the friction coefficient with sliding velocity (logarithmic *x*-axis) for high coverage all-atom and united-atom systems as well as experiment. Experimental data (*P_z_* = 0.69 GPa) are reproduced with permission from [48]. Dotted line extrapolation from point at which experimental data shows a constant gradient (5 × 10^4^ m·s^−1^) to sliding velocities accessible in NEMD simulations.

The results in section two describe differences between L-OPLS-AA (all-atom) and TraPPE-UA (united-atom) in terms of their description of the structure and friction of iron oxide surfaces, covered by stearic acid monolayers, confining a thin layer of *n*-hexadecane, during NEMD simulations. These results demonstrate the crucial role of force-field selection on the reproduction of experimental friction behavior in multicomponent tribological systems. Differences in the structure of the films obtained with the two force-fields are described first, followed by variations in their frictional behavior. The structures of the films as calculated with the two force-fields are very similar (Figure 4, Figure 5 and Figure 6); however, marked differences are visible in the system’s dynamic properties, such as the velocity profile (Figure 6) and the friction coefficient (Figure 7 and Figure 8). In what follows, some properties are expressed as functions of the distance from the surface, denoted by *z*; this is taken as the distance from the innermost layer of atoms on the bottom slab.

#### 3.2.1. Structure

Figure 4 shows the variation in the molecular tilt angle, θ, defined as the angle between a vector from the carbonyl carbon atom (C) to the terminal carbon atom (CTT) and the surface normal. The high coverage tilt angle of the stearic acid film agrees relatively well with values estimated from in situ AFM experiments (50° at 1.6 GPa) [57] for both the all-atom and united-atom force-fields. However, similar to previous MD simulations of thiol monolayers on gold [23], united-atom force-fields yielded reduced tilt angles, owing to the slightly lower area that each chain occupies, allowing molecules to lie flatter to the surface. The difference is most apparent at high coverage, where the molecules represented by the united-atom force-field are almost 5° more tilted than those represented by the all-atom force-field.

Figure 5 shows the ordering within the high coverage stearic acid films through separate radial distribution functions (RDFs), g(r), for the carbonyl carbon, C, and the terminal carbon, CTT. The C RDFs are shifted upwards by 10 units for clarity. RDFs from previous simulations have shown that the films move from liquid-like to amorphous to solid-like with increasing coverage [10]. The C RDFs show a large peak at *r* = 5 Å, which indicates ordering of the stearic acid molecules to the unit-cell dimension of the hematite surface [49]. There are also distinguishable C and CTT peaks at higher multiples of *r* = 5 Å indicating long-range order and a solidlike film. The united-atom and all-atom systems have very similar C and CTT RDFs, suggesting the united-atom force-field also yields a solid-like film at high coverage. The C peaks are better defined in the all-atom case, perhaps due to the non-polar hydrogens ‘locking’ the molecules in place and leading to a slightly more solid-like film.

The mass density profiles, *ρ*(*z*), in Figure 6 show the layering and interdigitation of the all-atom and united-atom systems. In order to uncover differences in the flow within all-atom and united-atom systems during sliding, velocity profiles, *v_x_*(*z*), are also shown. Atom velocities are computed for 0.5 Å spatial bins which are averaged over 100 ps time blocks during the sliding phase.

Sharp, intense peaks at the far left and right-hand side of the mass density profiles in Figure 6 indicate adsorption of carboxylic acid head groups on the surface, whilst the less intense peaks, which extend further from the surface, are due to the tail groups. The stearic acid films become substantially thicker and more structured with increasing coverage. Moreover, the level of interdigitation, indicated by the overlap of the solid and dotted lines, decreases with increasing coverage [10]. Figure 6 shows that the layering and interdigitation of the films are very similar between united-atom and all-atom models; however, the slab separation is slightly larger in the all-atom systems since the volume of each molecule is marginally greater than in the united-atom systems. The thickness of the stearic acid films themselves [57], and the system as a whole [58], agree well with experiment for both the all-atom and united-atom systems at high coverage.

The velocity profiles in Figure 6 indicate that there is no slip at the surface in any of the systems, as expected for the strongly absorbed head groups. The tail groups move at a similar velocity as the slab to which they are absorbed (±5 m·s^−1^) until the region where they become significantly interdigitated with the *n*-hexadecane lubricant. At this point, the velocity profile becomes Couette-like, indicating shear within the interdigitated region. The flow within the system changes slightly moving from an all-atom force-field to a united-atom force-field. For the all-atom system at high coverage, almost the entire stearic acid molecule moves with the wall, with multiple slip planes between the well-defined stearic acid and *n*-hexadecane layers. Although these layers seem equally well-defined in the united-atom mass density profile, the velocity profile has a shallower gradient and includes only one slip plane in the center of the system. This is more similar to the all-atom velocity profile at medium coverage. The low coverage velocity profile is more typical of conventional planar Couette flow, with a near linear velocity profile of the liquid between the slabs, rather than the region between the stearic acid tail groups. In the all-atom case, the low coverage velocity profile contains steps which indicate partial plug-flow between combined stearic acid-hexadecane layers [59]. These steps are less evident in the united-atom case, where the velocity profile is almost linear throughout the fluid. Collectively, the united-atom and all-atom velocity profiles suggest that the flow within the film changes depending on whether a united-atom or all-atom force-field is used. Generally, the flow is more Couette-like and there is less variation with coverage in the united-atom systems; specifically there is an absence of steps and more similar gradients in the velocity profiles.

#### 3.2.2. Friction

The influence of surface coverage on the friction coefficient was probed at a sliding velocity, *v*_s_ = 10.0 m·s^−1^ and a pressure, *P_z_* = 0.5 GPa (Figure 7a). The variation in the friction coefficient with sliding velocity (*v*_s_ = 1–20 m·s^−1^) was also examined at high, medium and low coverage and a pressure, *P_z_* = 0.5 GPa (Figure 7b). The high coverage friction-velocity behavior is then compared directly to experiments (Figure 8). The results indicate that the all-atom and united-atom systems yield considerably different friction behavior.

Friction-coverage behavior for the all-atom system is described first. At 10.0 m·s^−1^, the friction coefficient increases by 5% between low and medium coverage (Figure 7a); which can be rationalized as follows. In the liquid-like, low coverage system, there is a very high level of interdigitation (Figure 6) but molecular rearrangement is also fast, because the stearic acid molecules are widely spaced. This means that the shear stress is not significantly augmented as molecules from opposing slabs slide past one another. In the amorphous, medium coverage film, interdigitation is decreased; however, molecular rearrangement is much slower, due to stearic acid molecules being packed more closely; this results in increased shear stress and, thus, a higher friction coefficient [10].

In the all-atom system, the friction coefficient at high coverage is around 30% lower than at medium coverage, and approximately 25% less than at low coverage. Based on the structural changes with coverage, this reduction in friction can be attributed to the formation of clear slip planes between the stearic acid films and the *n*-hexadecane lubricant, as observed through atomic mass density and velocity profiles (Figure 6). These slip planes are facilitated through the close-packing of the tail groups, which leads to a solid-like, coherent monolayer film, which allows very little interdigitation with the *n*-hexadecane lubricant or with each another [10]. The increase in friction coefficient between low and medium coverage and then decrease between medium and high coverage (Figure 7b) observed at high velocity (*v*_s_ = 10.0 m·s^−1^) has been observed in AFM experiments of other surfactant films [60]. At low velocity (*v*_s_ = 1.0 m·s^−1^), the friction coefficient at medium coverage decreases to a value in between those at low coverage and high coverage (Figure 7b), since molecules have more time to rearrange relative to the sliding velocity [10]. Boundary friction experiments are always carried out at relatively low velocity, which rationalizes why a steady reduction in friction is observed when the concentration of stearic acid is increased [61].

In the united-atom systems, the structural changes with coverage are similar; however, the friction-coverage behavior is very different. Firstly, the friction coefficient is significantly lower in the united-atom systems for all of the coverages simulated. This is probably due to the artificial smoothness of the hydrogen-free united-atom molecules which leads to a reduction in the steric barriers to interfacial sliding. Moreover, the friction-coverage trend (Figure 7a) is the opposite of that observed in the all-atom NEMD simulations as well as AFM [60] and boundary friction experiments [48]. In the all-atom systems, in accordance with experiment, increasing coverage leads to reduced interdigitation and a significant reduction in the friction coefficient. This behavior is not replicated for united-atom systems, since the smoother molecules can slide over one another more easily, meaning that the level of interdigitation has less influence on the friction coefficient. As a result, the increased viscous contribution to friction from the thicker, high coverage film becomes more significant than any reduction due to slip plane formation. Therefore the united-atom friction coefficient increases with increasing coverage of stearic acid, despite the reduced interdigitation.

It is clear from the fitting curves in Figure 7b that the friction coefficient increases linearly with the logarithm of the sliding velocity for the all-atom and united-atom systems at all coverages. This is in accordance with experimental results and stress-promoted thermal activation theory [48]. However, the friction coefficient is much lower in the united-atom systems; the greatest difference (−45%) is at low and medium coverage, and the smallest difference (−30%) is at high coverage, where there is significantly less interdigitation. This further suggests that the contribution to friction due to interdigitation is severely under-predicted when using a united-atom force-field. The rate of increase in friction coefficient with increasing sliding velocity is also slightly less for united-atom systems and there is less variation between the different coverages. For example, the steeper increase in the friction coefficient evident in the medium coverage all-atom system relative to the other coverages is not as prominent in the united-atom system.

Figure 8 shows that the friction coefficients from the high coverage all-atom system agrees extremely well with extrapolations from boundary friction experiments [48] conducted at lower sliding velocities (3.0 × 10^−7^–5.0 × 10^−3^ m·s^−1^), whereas united-atom friction coefficients are around 30% lower than these extrapolations. The linear extrapolation of the experimental data to higher sliding velocities is justified since this linear increase in the friction coefficient with the logarithm of sliding velocity is predicted by stress-promoted thermal activation theory [48]. The all-atom simulation data (boundary thermostatted to 300 K) appear to show slightly better agreement with the experimental data collected at 373 K than data collected at 308 K. Experimentally, the temperature may influence both the level of interdigitation and speed of molecular rearrangement within the films, as well as the surface coverage [48]. Since the exact coverage is not known in the experimental systems, it is not possible to determine the relative significance of these factors on the friction coefficient; this could be investigated in future simulations where coverage and temperature are varied independently.

An under-prediction in the friction coefficient when using united-atom force-fields is evident from the comparison to experiment shown in Figure 8. This under-prediction is expected to be relevant in all NEMD simulations of long-chain, linear molecules, and will be particularly severe when the molecules are densely packed but not clearly layered, for example the medium coverage films in these simulations (Figure 7b). In line with the viscosity [3], friction under-prediction is expected to be much more severe for linear molecules, than those with significant branching.

## 4. Summary and Conclusions

In order to be confident that behavior observed in NEMD simulations is representative of real dynamical systems, the selection of an accurate force-field is essential. In this study, a wide range of popular force-fields have been benchmarked for their density and viscosity prediction of *n*-hexadecane under ambient and HTHP conditions. Significant issues with many popular force-fields were highlighted for the simulation of long-chain, linear alkanes under both ambient and HTHP conditions. Specifically, all of the united-atom force-fields under-predicted the viscosity, and the original parameterizations of all-atom force-fields resulted in an elevated melting point for *n*-hexadecane, leading to anomalous density and viscosity values. Both of these problems are expected to significantly affect the behavior in confined NEMD simulations. The most accurate force-field under the conditions tested was L-OPLS-AA, which was specifically parameterized for long-chain molecules.

The most effective united-atom (TraPPE-UA) and all-atom (L-OPLS-AA) force-fields were then applied in confined NEMD simulations of a test system. In particular, a multicomponent tribological system where high temperatures and pressures are especially relevant, was chosen in order to compare the differences between the force-fields in terms of their prediction of structure and friction. Both the all-atom and united-atom force-fields accurately reproduced the experimental structure of stearic acid films adsorbed on iron oxide surfaces and separated by a thin layer of *n*-hexadecane. However, differences are observed in the flow within the films under sliding, with steeper, more stepped velocity profiles for the all-atom systems. The friction behavior is also very different between the all-atom and united-atom systems. In the all-atom systems, friction generally decreases with increasing coverage, in accord with experimental results. This is due to reduced interdigitation and the formation of clear slip planes between the stearic acid and *n*-hexadecane layers. In the united-atom systems, the friction coefficient is much lower, and generally increases with increasing coverage, suggesting that interdigitation is less critical to the friction coefficient, which appears to be dominated by the viscous contribution due to shearing within the film. United-atom force-fields are able to reproduce the linear increase with the logarithm of sliding velocity predicted by stress-promoted thermal activation theory and captured by all-atom simulations and experiments. However, while the high coverage all-atom results fit extremely well with experimental extrapolations, the friction coefficient is under-predicted by 30% in the united-atom system.

The results from the first section suggest that an accurate, all-atom force-field, such as L-OPLS-AA is required in order to accurately predict the experimental viscosity of long-chain molecules. Moreover, results from the second section indicate that experimental friction behavior of confined, multi-component tribological systems is only captured using an accurate, all-atom force-field.

United-atom force-fields have been, and will likely continue to be, useful to capture trends in tribological simulations of very large, complex systems due to their relatively low computational expense. However, systematic under-prediction of the viscosity and friction of long-chain, linear molecules make them less accurate for NEMD simulations of tribological systems. Thus, it is recommended that all-atom force-fields, specifically L-OPLS-AA, are used in future MD simulations of systems containing long-chain, linear molecules, where computational resources allow.
